# Explainable Machine Learning Applied to Bioelectrical Impedance for Low Back Pain: Classification and Pain-Score Prediction

**DOI:** 10.3390/s25196135

**Published:** 2025-10-03

**Authors:** Seungwan Jang, Seung Mo Yoo, Se Dong Min, Changwon Wang

**Affiliations:** 1 Department of Software Convergence, Soonchunhyang University, Asan 31538, Republic of Korea; seungwanjang93@gmail.com; 2 Occupational and Environmental Medicine, Yesan Myongji Hospital, Yesan 32423, Republic of Korea; 3 Department of Medical IT Engineering, Soonchunhyang University, Asan 31538, Republic of Korea; 4 Department of Integrated Healthcare Science, Soonchunhyang University, Asan 31538, Republic of Korea

**Keywords:** bioelectrical impedance, explainable machine learning, low back pain, SHAP, XGBoost

## Abstract

(1) Background: Low back pain (LBP) is the most prevalent cause of disability worldwide, yet current assessment relies mainly on subjective questionnaires, underscoring the need for objective and interpretable biomarkers. Bioelectrical impedance parameter (BIP), quantified by resistance (R), impedance magnitude (Z), and phase angle (PA), reflects tissue hydration and cellular integrity and may provide physiological correlates of pain; (2) Methods: This cross-sectional study used lumbar BIP and demographic characteristics from 83 participants (38 with lumbar BIP and 45 normal controls). We applied Extreme Gradient Boosting (XGBoost), a regularized tree-based machine learning (ML) algorithm, with stratified five-fold cross-validation. Model interpretability was ensured using SHapley Additive exPlanations (SHAP), which provide global importance rankings and local feature attributions. Outcomes included classification of LBP versus healthy status and regression-based prediction of pain scales: the Visual Analog Scale (VAS), Oswestry Disability Index (ODI), and Roland–Morris Disability Questionnaire (RMDQ); (3) Results: The classifier achieved high discrimination (ROC–AUC = 0.996 ± 0.009, sensitivity = 0.950 ± 0.068, specificity = 0.977 ± 0.049). Pain prediction showed best performance for VAS (R^2^ = 0.70 ± 0.14; mean absolute error = 1.23 ± 0.27), with weaker performance for ODI and RMDQ; (4) Conclusions: These findings suggest that explainable ML models applied to BIP could discriminate between LBP and healthy groups and could estimate pain intensity, providing an objective complement to subjective assessments.

## 1. Introduction

Low back pain (LBP) remains a major cause of disability and healthcare use worldwide, resulting in significant personal and social costs [1,2]. In daily practice, assessment relies on clinical examination and self-reported questionnaire results such as Visual Analog Scale (VAS), Roland–Morris Disability Questionnaire (RMDQ), and Oswestry Disability Index (ODI) [2,3]. While these instruments are indispensable, they capture symptoms rather than organizational characteristics, and since they rely on patient-subjective reporting, outcomes may vary depending on individual psychological conditions, understanding, and cultural differences [2,4]. To compensate for these limitations, there is a persistent need for objective, non-invasive biomarkers that reflect the biophysical state of lumbar tissues and complement symptom-based assessment [5,6].

Bioelectrical impedance parameter (BIP) analysis can provide objective information to evaluate tissue conditions related to hydration, cell integrity, and cell membrane properties by measuring and quantifying tissue electrical properties (Resistance (R), Impedance (Z), phase angle (PA), and Capacitance (C) [5,6,7]. Prior work [8] has explored BIP for LBP assessment using statistical comparisons and cut-off-based rules, showing promising group-level differences. However, purely statistical thresholds struggle to accommodate non-linear effects, complex interactions (e.g., between resistance and phase angle), and potential left–right asymmetry at the lumbar region. Machine learning approaches to pain biomarkers have not yet incorporated BIP features, although other studies have analyzed cortical thickness and functional connectivity (rs-fMRI) to elucidate neural mechanisms of pain, and some have predicted LBP using secondary data, sEMG, and motion capture [9,10,11]. Research applying machine learning (ML) to LBP has otherwise relied mainly on non-electrical features such as survey-based psychosocial data [12] or trunk kinematic patterns in postpartum women [13]. To our knowledge, the integration of localized lumbar BIP parameters into an explainable ML framework has been highly limited. This study addresses that gap by combining BIP indices with demographic variables in an Extreme Gradient Boosting (XGBoost) model, enabling both LBP–healthy discrimination and pain-score prediction.

Gradient-boosted decision trees, and the XGBoost in particular, are well-suited to tabular biomedical data that combine anthropometric and biophysical variables [14]. They model non-linearities and higher-order interactions, include explicit regularization (L1/L2), and use row/column subsampling to control variance—features that are advantageous when sample size is modest relative to the number of predictors [15]. At the same time, explainability is essential: clinicians require not only accurate predictions but also a clear rationale that aligns with physiology [16]. SHapley Additive exPlanations (SHAP) provide global importance rankings and local attributions, enabling inspection of conditional patterns and interactions without altering the trained model [16].

To date, most applications of machine learning in LBP have relied on non-electrical features such as psychosocial questionnaires [12] or trunk kinematic patterns [13]. More recently, explainable ML has also been applied to other modalities, for example, MRI radiology reports and EMR data for lumbar disk herniation [17], highlighting the importance of interpretability for clinical adoption. Our previous study [8] demonstrated that localized lumbar BIP parameters could distinguish LBP from healthy groups using conventional statistical analyses, but this approach did not extend to individual-level prediction or capture feature interactions.

This study bridges group-level BIP differences and patient-level decision support by unifying LBP classification and pain-score prediction in a single, explainable framework. By quantifying which BIP indices and anthropometric measures drive model decisions—and how their interactions shape risk—the work aims to advance objective, clinically interpretable assessment of LBP and lay groundwork for prospective validation and integration into routine workflows.

## 2. Methods

### 2.1. Dataset

The present study used a dataset from a cross-sectional analysis study previously collected in our earlier study [8], which included 85 participants (healthy: *n* = 45; LBP: *n* = 40) enrolled between 2023 and 2024 at Yesan Myongji Hospital, Republic of Korea (Table 1). That study complied with the Declaration of Helsinki and was approved by the Institutional Review Board of Soonchunhyang University (protocol code: 1040875-202303-SB-016). The dataset comprised demographic and anthropometric variables (sex, height, weight, waist circumference, spinal length), BIP (resistance, impedance magnitude, phase angle, capacitance) measured bilaterally, and patient-reported pain scores (VAS, ODI, RMDQ).

Measurements were performed using the Pain Bot device (Red & Blue Co., Ltd., Yesan, Republic of Korea), a certified Class II medical combinational stimulator, which employs a bipolar configuration with a probe supplying alternating current (AC) and an electrode for measurement. The system operates at 182 Hz with a 50 mA output current. To minimize any potential therapeutic effects, the output voltage was set at 3.5 V in continuous pulse mode, and each trial was limited to 15 s. Participants were positioned prone, and standardized Ag/AgCl electrodes and probes were applied 50 mm laterally from the L5 and L1 spinous processes, respectively. Each side (left and right) was measured three times per trial, with 30 s rest between trials, yielding six measurements per participant.

### 2.2. Bioelectrical Impedance Parameter Calculation

Resistance (R) was calculated based on Ohm’s law, and tissue-specific permittivity values together with sex-based tissue composition ratios were applied to calculate the overall permittivity ($\varepsilon_{r}$). These values were determined based on established dielectric property data of human tissues [18] and had already been applied in our previous study [8]. Using a cylindrical model of the waist between L1 and L5, the cross-sectional area (A) and electrode distance (D), along with the vacuum permittivity ($\varepsilon_{0}$), were used to estimate capacitance (C = $\varepsilon_{0}\varepsilon_{r}\frac{A}{D}$), from which impedance (Z = $\sqrt{\frac{1}{\frac{1}{R^{2}}+{\left(2\pi fC\right)}^{2}}}$) and phase angle (PA = −Arctan(2πfCR)) were derived. The adopted values are summarized in Table 2.

### 2.3. Data Preprocessing

Two participants were excluded due to incomplete data (one subject with missing left-side bioelectrical impedance measurements and another with missing demographic information), resulting in a final dataset of 83 subjects (healthy: *n* = 45; LBP: *n* = 38). Continuous features—including demographic measures (height, weight, waist circumference, spinal length) and bioelectrical impedance parameters (right/left R, Z, PA, and C)—were standardized to z-scores. The binary variable, sex, was retained as a 0/1 indicator without scaling. Left and right lumbar measurements were treated as distinct predictors rather than averaged to preserve potential lateral asymmetry (e.g., R_right and R_left). No additional imputation or resampling was performed. To avoid information leakage, all transformations were fit on the training split within each cross-validation fold and applied to the corresponding validation split only. Table 3 shows the feature list used in this study.

### 2.4. Rationale for Model Choice and Explainability

We selected XGBoost as the primary learner because gradient-boosted trees have repeatedly shown strong, state-of-the-art performance on tabular biomedical data, where heterogeneous predictors and non-linear effects are common. XGBoost natively captures non-linearities and higher-order interactions and is robust on medium-sized tabular datasets relative to deep neural networks [19]. In addition, the algorithm incorporates explicit regularization (L1/L2), shrinkage via the learning rate, and row/column subsampling, which help control variance and mitigate overfitting—an important consideration when the number of subjects is modest compared with the number of features [20]. Tree-based ensembles also avoid strong distributional assumptions, provide probabilistic outputs under the logistic objective, and are computationally efficient, enabling reproducible evaluation and downstream analyses.

In this study, we developed two complementary, explainable ML models grounded in BIP and demographic/anthropometric features. First, we built an XGBoost classifier to discriminate LBP from healthy status, evaluating performance with stratified 5-fold cross-validation and interpreting feature contributions using SHAP values (summary and dependence plots) to probe physiologically plausible interactions. Second, using the same prespecified predictor set and preprocessing, we trained XGBoost regressors to predict continuous pain outcomes—VAS, ODI, and RMDQ—and summarized performance with MAE, RMSE, R^2^, and Spearman’s ρ.

For clinical adoption, accuracy alone is not sufficient; models should be interpretable, auditable, and physiologically coherent to support trust, safety, and accountability [21]. Therefore, we paired XGBoost with SHAP to quantify how predictors contribute to decisions at both the global (population-level importance) and local (individual-level attribution) scales. SHAP provides a unified, axiomatically grounded framework for feature attributions, with efficient TreeSHAP algorithms and tools that aggregate local attributions into global summaries and feature-interaction views [22].

### 2.5. LBP Classification Model

In this study, the XGBoost model was developed to discriminate LBP from healthy status. Hyperparameters were optimized via Bayesian optimization (Optuna Version 4.0.0), using the area under the receiver operating characteristic curve (ROC-AUC) as the optimization objective. The search space included n_estimators, max_depth, learning_rate, subsample, colsample_bytree, gamma, reg_alpha, and reg_lambda (bounded as follows: n_estimators 100–500, max_depth 3–8, learning_rate 0.01–0.3 [log scale], subsample 0.6–1.0, colsample_bytree 0.6–1.0, gamma 0–5, reg_lambda 1 × 10^−3^–10 [log scale], reg_alpha 1 × 10^−3^–10 [log scale]). After hyperparameter tuning, model performance was estimated with 5-fold stratified cross-validation. For each fold, we computed accuracy, precision, recall (sensitivity), specificity, F1-score, and ROC-AUC. Given the modest sample size (N = 83), we adopted 5-fold stratified cross-validation with a fixed random seed to enhance robustness and to preserve class proportions in every fold. Stratification ensures each fold adequately represents the overall class distribution, supporting reliable estimation of discrimination and error rates. The final model used the Optuna-derived hyperparameters for training and prediction. All analyses were conducted in Python Version 3.7.1 using scikit-learn, XGBoost, Optuna, and SHAP.

SHAP values were computed on the tuned XGBoost model to quantify global and local feature contributions. SHAP was used for interpretation only—the classifier was trained on the full, prespecified predictor set without any SHAP-based feature selection or model refitting. Specifically, we used SHAP to (i) rank influential features driving LBP discrimination, (ii) visualize conditional and interaction effects via up to five prespecified dependence plots (x-axis: feature value; y-axis: SHAP value; color: interacting feature), and (iii) assess clinical plausibility while screening for spurious associations. The exact feature pairs examined in the dependence plots are reported in the Results.

### 2.6. Pain Score Prediction Model

We developed prediction models for VAS, ODI, and RMDQ using BIP and anthropometric/demographic features. To ensure comparability with the classification pipeline, we implemented XGBoost regression with the same prespecified predictor set and identical preprocessing applied in classification. In the primary specification, we reused the Optuna-tuned classification hyperparameters and changed the objective to reg:squarederror. As a sensitivity analysis, we performed outcome-specific Bayesian optimization (Optuna) to minimize RMSE (internal 3-fold CV; search space: n_estimators, max_depth, learning_rate, subsample, colsample_bytree, gamma, reg_alpha, reg_lambda). Predictive performance was estimated with 5-fold cross-validation (fixed random seed), reporting MAE, RMSE, R^2^, and Spearman’s ρ; for VAS, we additionally reported the proportions within ±1 and ±2 points. For visual assessment, we produced predicted-versus-observed scatter plots (identity line; VAS additionally with ±1/±2 bands) and paired index-wise plots linking each observed values to its prediction to display residuals.

## 3. Results

### 3.1. LBP Classification Performance

Hyperparameters of the XGBoost model were optimized via Bayesian optimization using the ROC-AUC score. A representative best configuration was max_depth = 5, n_estimators = 409, learning_rate = 0.162, subsample = 0.867, colsample_bytree = 0.607, gamma = 4.478, reg_alpha = 0.012, and reg_lambda = 0.060 as shown in Table 4.

**Figure 1:**
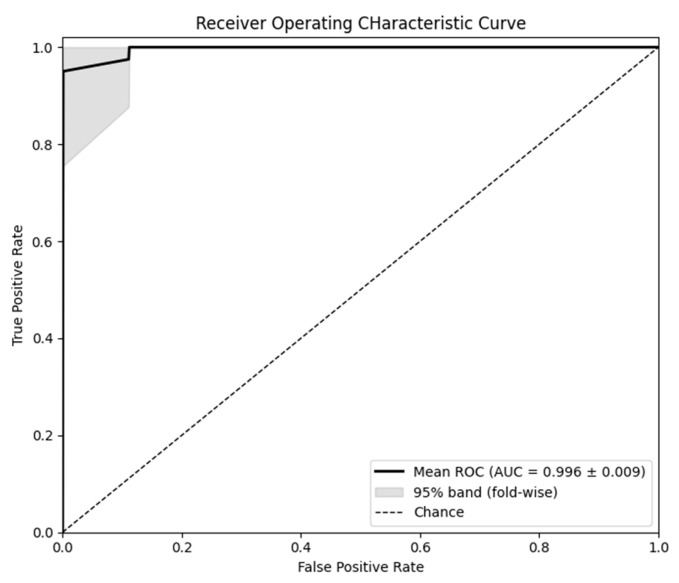
Receiver operating characteristic (ROC) curves across the 5 cross-validation folds for the tuned XGBoost model.

Across the 5-folds, the tuned XGBoost achieved accuracy 0.952 ± 0.049, precision 0.975 ± 0.055, recall 0.925 ± 0.068, specificity 0.977 ± 0.049, F1-score 0.948 ± 0.052, and ROC-AUC 0.996 ± 0.009, indicating consistently high discrimination with balanced sensitivity and specificity. The mean ROC curve with 95% band shading across folds is shown in Figure 1.

### 3.2. Confusion Matrices and Subgroup Performance

**Figure 2:**
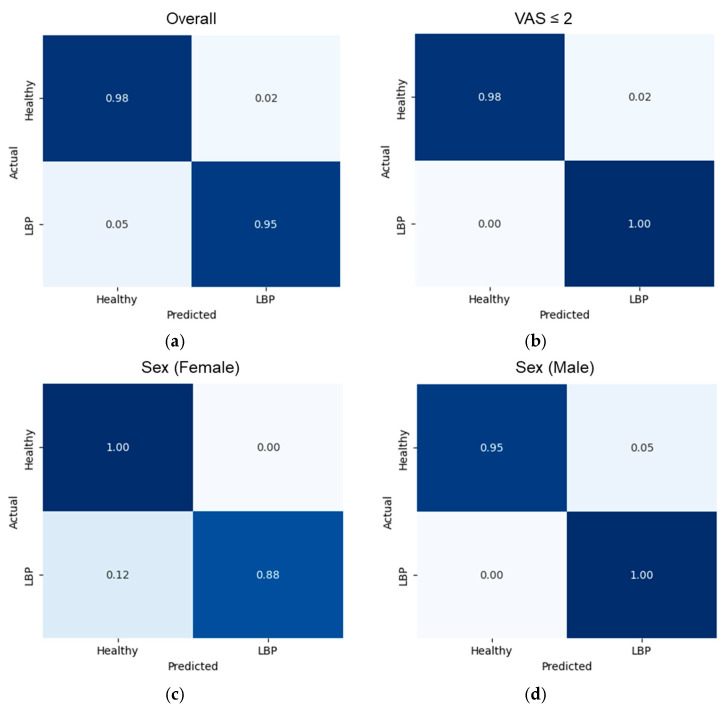
Confusion matrices healthy vs. BLP classification. (**a**) Overall, (**b**) VAS ≤ 2, (**c**) sex (female), (**d**) sex (male).

Comparing healthy and LBP groups, the overall confusion matrix showed balanced performance with high true-negative and true-positive counts (TN = 44, FP = 1, FN = 2, TP = 36), corresponding to row-normalized specificity = 0.98, sensitivity = 0.95, precision = 0.97, F1 = 0.96, and AUC = 0.992. Sex-stratified matrices exhibited similar patterns without evidence of systematic bias. Among females (TN = 26, FP = 0, FN = 2, TP = 15), specificity was 1.00 and sensitivity 0.88, with precision = 1.00, F1 = 0.94, and AUC = 0.998. Among males (TN = 18, FP = 1, FN = 0, TP = 21), specificity was 0.95 and sensitivity 1.00, with precision = 0.96, F1 = 0.98, and AUC = 0.991. Stratification by pain intensity showed that only the VAS ≤ 2 stratum contained both classes; in this subset (TN = 44, FP = 1, FN = 0, TP = 1), specificity was 0.98 and sensitivity 1.00, with precision = 0.50, F1 = 0.67, and AUC = 1.00—metrics that should be interpreted cautiously given the single positive case. The intermediate (2 < VAS ≤ 4) and higher (VAS > 4) strata contained a single observed class and were therefore not reported, see Figure 2.

### 3.3. Baseline by Feature Group

**Figure 3:**
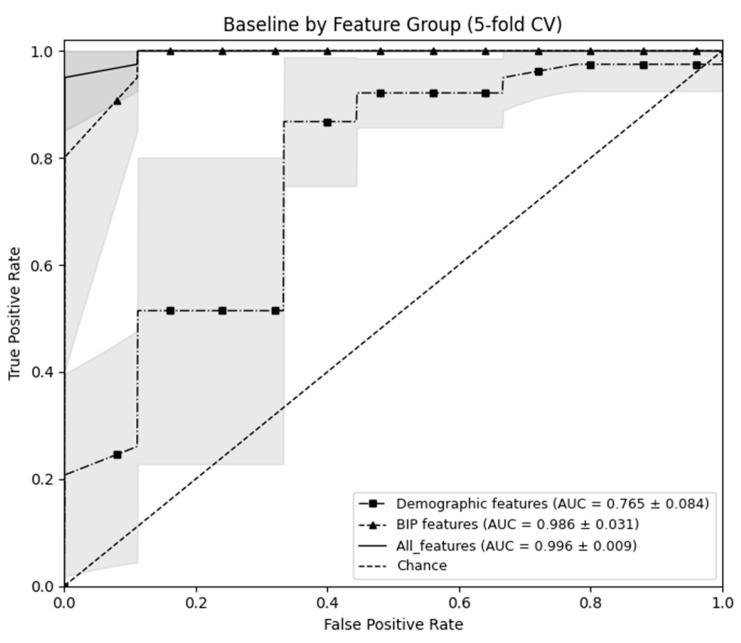
Mean ROC curve by predictor set (Shading indicates the SD of AUC across cross-validation folds).

To probe potential overfitting and isolate the incremental value of BIP, we evaluated three predefined feature sets using stratified 5-fold cross-validation: demographic-only, BIP-only, and All (demographic + BIP). The demographic baseline yielded modest discrimination (ROC-AUC 0.765 ± 0.083, accuracy 0.733 ± 0.103), whereas the BIP-only model achieved substantially higher performance (ROC-AUC 0.986 ± 0.031, accuracy 0.964 ± 0.052, sensitivity 0.950 ± 0.068, specificity 0.977 ± 0.049). Combining BIP with demographics produced the best ROC-AUC (0.996 ± 0.009), with balanced error rates, indicating that (i) most of the discriminative signal resides in BIP and (ii) demographics provide complementary—but smaller—gains (Table 5 and Figure 3).

### 3.4. Key Indicators for LBP Classification

**Figure 4:**
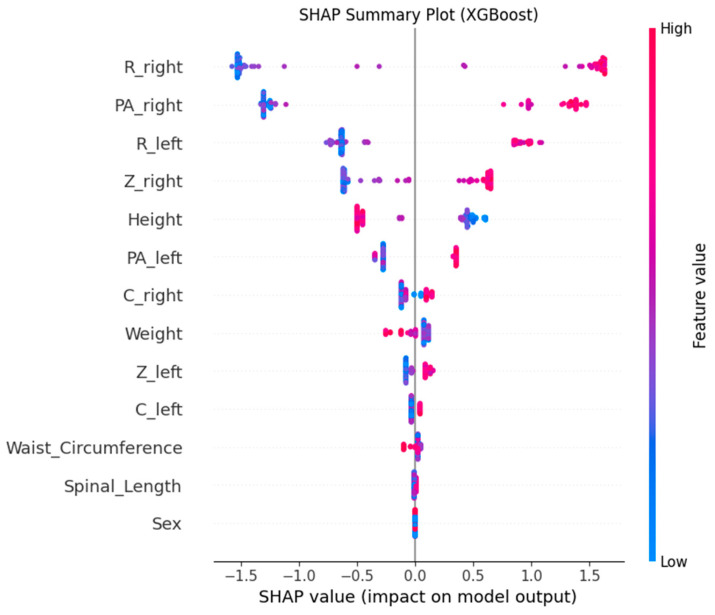
SHAP summary plot showing global feature contributions.

To interrogate the global contribution of predictors, SHAP values were computed on the tuned XGBoost model. Figure 4 indicated that R_right, PA_right, R_left, Z_right, and Z_left were among the top contributors by mean |SHAP|. Collectively, these findings suggest that electrical resistance/impedance and phase angle—especially on the right side—encode clinically relevant information for LBP discrimination. From a physiological perspective, higher resistance (R) is consistent with altered fluid distribution and tissue properties; impedance magnitude (Z) reflects composite conductive/resistive changes; and lower phase angle (PA) is often associated with reduced cell membrane integrity and poorer cellular health.

**Figure 5:**
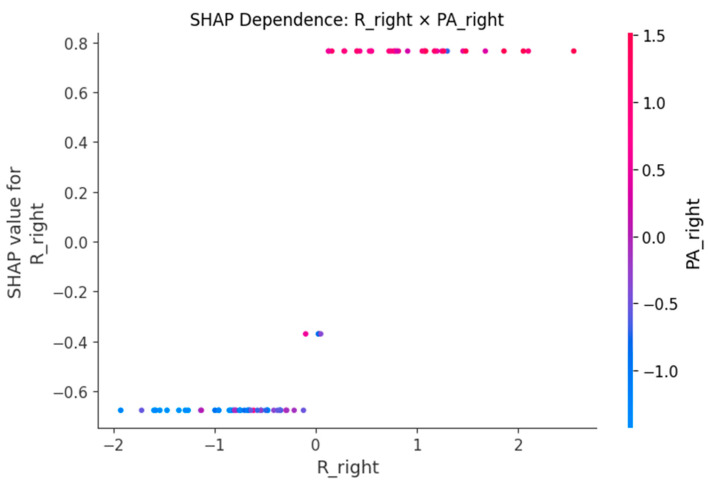
SHAP dependence plot for R_right with PA_right as the interaction.

**Figure 6:**
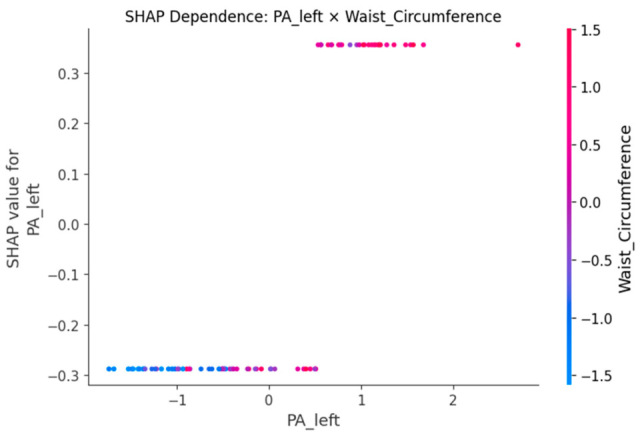
SHAP dependence plot for PA_left with Waist_Circumference as the interaction.

**Figure 7:**
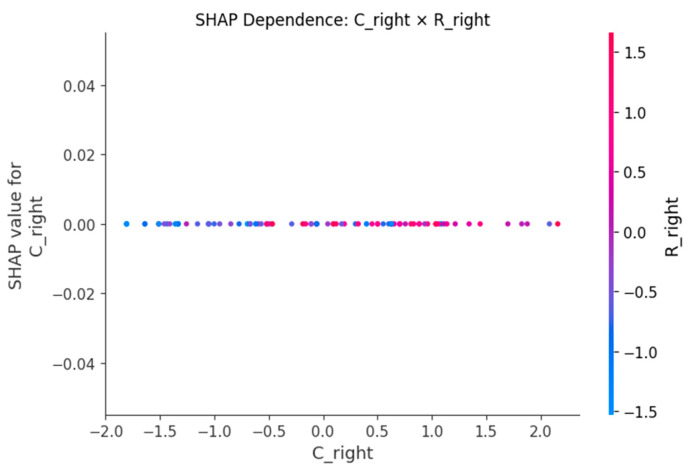
SHAP dependence plot for C_right with R_right as the interaction.

**Figure 8:**
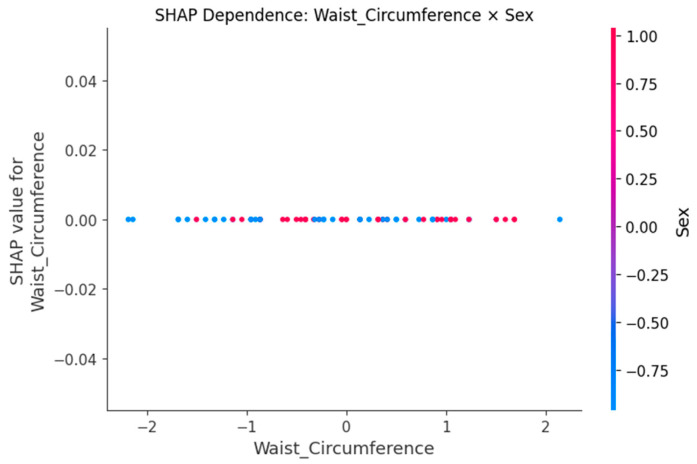
SHAP dependence plot for Waist_Circumference with sex as the interaction.

**Figure 9:**
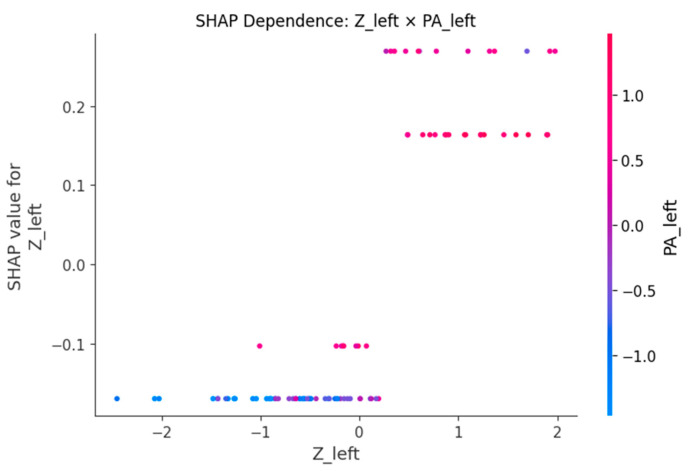
SHAP dependence plot for Z_left with PA_left as the interaction.

SHAP dependence plots (Figure 5, Figure 6, Figure 7, Figure 8 and Figure 9) were used to visualize how variation in individual predictors—and their interactions encoded by color—shapes model output (probability of LBP). Increasing R_right was associated with higher SHAP values, indicating greater LBP probability, and this effect was amplified when PA_right was low (Figure 5). Lower PA_left contributed positively to LBP classification, with a stronger effect among participants with larger waist circumference (or higher C_left) (Figure 6). Higher C_right was likewise linked to increased LBP probability, particularly when R_right was concurrently high (Figure 7). Greater waist circumference generally increased LBP probability and exhibited sex-specific slopes, suggesting modulation by sex-related differences in fat distribution and muscle mass (Figure 8). Finally, higher Z_left contributed positively to LBP classification, with more pronounced effects at lower PA_left (or higher R_left) (Figure 9).

### 3.5. VAS, ODI, RMDQ Score Prediction Performance

Using 5-fold cross-validation, XGBoost regression predicted VAS, ODI, RMDQ scores with the following performance as shown in Table 6.

**Figure 10:**
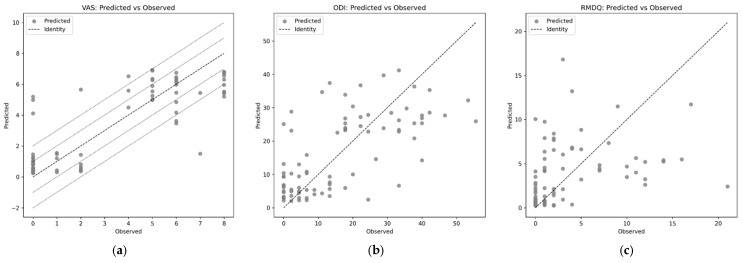
Predicted vs. observed VAS, ODI, RMDQ scores, (**a**) VAS (the black dashed line denotes the identity line, representing perfect agreement, and the black dotted bands indicate ±1 and ±2 VAS units), (**b**) ODI, (**c**) RMDQ.

**Figure 11:**
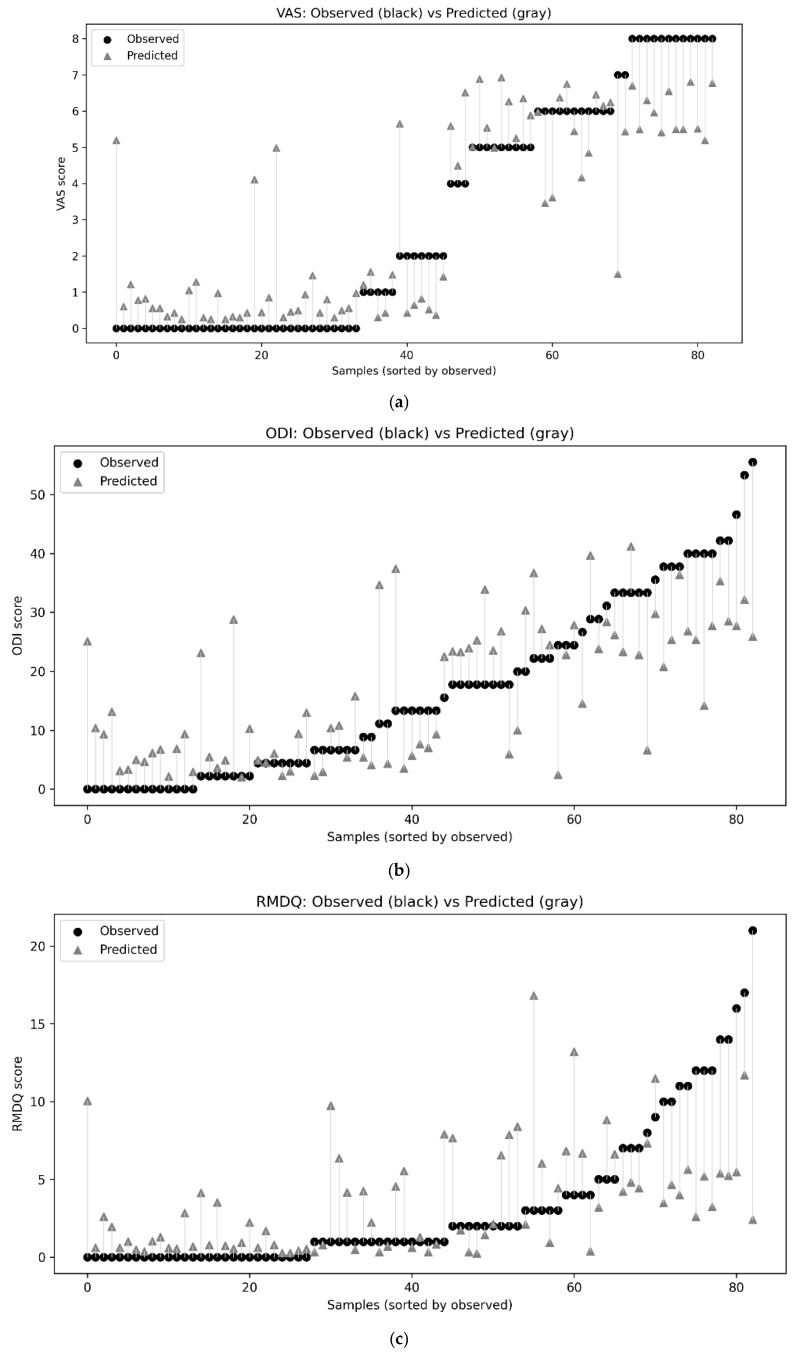
Paired index-wise comparison of observed and predicted scores, (**a**) VAS, (**b**) ODI, (**c**) RMDQ.

For VAS, the model achieved MAE 1.229 ± 0.268, RMSE 1.641 ± 0.414, R^2^ 0.702 ± 0.14, and Spearman’s ρ 0.787 ± 0.082; additionally, 55.5% ± 5.9% and 81.8% ± 7.8% of predictions fell within ± 1 and ± 2 points, respectively. For ODI, performance was MAE 8.975 ± 1.311, RMSE 11.312 ± 2.00, R^2^ 0.330 ± 0.377, and ρ 0.650 ± 0.141. For RMDQ, performance was MAE 3.239 ± 0.626, RMSE 4.637 ± 1.087, R^2^ −0.087 ± 0.373, and ρ 0.585 ± 0.232. Overall, VAS showed the most reliable predictability with moderate-to-strong correlation and reasonable absolute error, whereas ODI yielded modest explained variance and RMDQ predictions showed negative explained variance, indicating failure beyond baseline performance, as shown in Figure 10 and Figure 11.

## 4. Discussion

This study demonstrates that BIP and demographic features can support two complementary and explainable ML tasks in LBP: (i) discrimination of LBP vs. healthy status and (ii) prediction of pain scores (VAS, ODI, RMDQ). Among conventional models, Logistic Regression (LR) achieved an AUC of 0.972 ± 0.00, while Random Forest (RF) and Support Vector Machine (SVM) both yielded 0.944 ± 0.01. XGBoost, however, outperformed these approaches, delivering the highest discrimination with a cross-validated ROC-AUC of 0.996 ± 0.009, balanced sensitivity of 0.950 ± 0.068, and specificity of 0.977 ± 0.049 (Table 7).

Our results are consistent with broader evidence that LBP imposes a major global burden and that objective, scalable tools are needed to complement symptom scales [1,23].

To address potential overfitting, we compared predefined predictor sets in a baseline analysis (Table 5; Figure 3). A demographic-only model performed modestly (ROC-AUC ~0.77), whereas a BIP-only model reached ROC-AUC ~0.99 with balanced sensitivity and specificity, indicating that discrimination is driven by genuine signal in the impedance features rather than spurious fit. Adding demographics to BIP yielded only a small incremental gain (ROC-AUC 0.986 → 0.996), consistent with BIP as the dominant contributor and demographics as complementary context. SHAP findings (higher R, lower PA with interpretable interactions), these results support the physiological plausibility and robustness of the learned decision rules.

BIP features are clinically meaningful because their constituent measures R, Z and PA encode tissue hydration and cellular integrity. Lower PA is repeatedly associated with impaired cellular health and inflammatory states, which supports our finding that low PA increases the probability of LBP [24,25]. In an LBP-specific cohort [8], BIP-based analyses also reported group differences and links to disability, aligning with our SHAP-derived importance of R and PA. Mechanistically, previous studies have shown that higher R is generally associated with reduced fluid content or altered tissue composition [18], while lower PA has been repeatedly linked to diminished cell membrane integrity and inflammatory states [24,25]. SHAP analyses consistently highlighted higher resistance (R) and lower phase angle (PA) as increasing LBP probability, with lateral (left–right) and conditional effects. The dependence plots further reveal interpretable interactions. The amplification of resistance effects when PA is low (R_right × PA_right) echoes the coupling between membrane integrity and conductive pathways [26]. The stronger impact of low PA at larger waist circumference suggests an adiposity–inflammation context that modulates electrical signatures, in line with literature linking PA to inflammatory burden [24,27]. The C_right × R_right pattern (high capacitance plus high resistance) is compatible with hydration/membrane-related changes seen in impedance work, while the Z_left × PA_left effect indicates that global impedance becomes more informative as PA declines [28]. Finally, preserving left–right features rather than averaging likely aided detection of lateral phenomena; asymmetry and paraspinal muscle quality have been associated with clinical outcomes in LBP populations, supporting the plausibility of side-specific signals [29,30]. Methodologically, pairing XGBoost for non-linear, interaction rich tabular data with SHAP for post hoc global/local attributions and interaction views follows best practice in explainable ML and facilitates transparent, clinician-facing interpretation [20,31].

Regarding the pain-score prediction models, VAS was the most predictable target (MAE: 1.229 ± 0.268; RMSE: 1.641 ± 0.414; R^2^: 0.702 ± 0.140; ρ: 0.787 ± 0.082). In contrast, ODI scores showed moderate predictive performance (MAE: 8.975 ± 1.311; RMSE: 11.312 ± 2.00; R^2^: 0.330 ± 0.377; ρ: 0.650 ± 0.141), and RMDQ scores were the most challenging to predict (MAE: 3.229 ± 0.626; RMSE: 4.637 ± 1.87; R^2^: −0.087 ± 0.373; ρ: 0.585 ± 0.232). In our previous study [8], we relied solely on group-level statistical analyses of correlations between BIP and pain scores (VAS, ODI, RMDQ); in contrast, the current study applied an explainable ML framework to enable both classification and prediction. This divergence is explained by the fact that VAS reflects contemporaneous pain intensity, whereas ODI and RMDQ quantify broader functional impact shaped by behavioral and psychosocial factors that BIP alone may not capture [23,32,33]. In addition, differences in scoring systems and potential cultural- or language-dependent interpretations may weaken correspondence between biophysical measures and pain questionnaires [2]. These results indicate that BIP-derived indices are valuable as objective biomarkers of LBP intensity but have inherent limitations in predicting multidimensional disability scores. This interpretation aligns with our previous study [8], which also emphasized the stronger correspondence between BIP and LBP intensity compared with complex psychosocial outcomes. Therefore, BIP should not be considered a substitute for psychosocially oriented instruments such as ODI and RMDQ, but rather as a complementary modality. Future studies should explore multimodal approaches integrating BIP with imaging, wearable activity monitoring, and validated psychosocial assessments to more comprehensively capture the biopsychosocial spectrum of LBP.

Research on employing bioimpedance in the context of low back pain has been conducted, mainly focusing on body composition or correlation-based analyses. However, the application of ML methods to BIP (R, Z, PA) has been highly limited, with most existing ML approaches relying instead on non-electrical features such as survey or motion data [12,13]. The present study therefore represents a novel extension by integrating localized lumbar BIP indices into an explainable ML framework, enabling both discrimination and pain-score prediction.

Clinically, these findings may support BIP as an objective adjunct to symptom-based assessment. The classifier could aid screening/triage or risk stratification, while VAS prediction may assist follow-up and treatment monitoring when direct reporting is unavailable or noisy. SHAP summary rankings identify which BIP/anthropometric factors drive decisions, and dependence plots expose interaction regimes that align with known physiology. Importantly, SHAP was used strictly for interpretation (not feature selection), reducing optimistic bias and preserving a transparent predictor–outcome link.

This study is strengthened by an explainable gradient-boosted architecture tailored to structured biomedical data, a prespecified predictor set, rigorous control of information leakage across folds, stratified 5-fold cross-validation, explicit reproducibility safeguards (consistent preprocessing), and a unified pipeline spanning classification and regression. Several limitations must be acknowledged.

First, the present analysis reuses a dataset previously collected at a single center, so the sample size is fixed, and no external cohort was available for validation. Nevertheless, our earlier study already demonstrated robust discrimination between LBP and healthy groups using conventional ROC analyses (AUC > 0.96 for R, Z, and PA). The current work extends those findings by applying machine learning, which further improved performance by capturing complex feature interactions. These results suggest that lumbar bioimpedance provides a strong physiological signal and indicates its potential utility as an objective biomarker for LBP.

Second, the analysis relied on a single frequency (182 Hz). While this frequency had previously shown sufficient discrimination between groups, it mainly reflects extracellular matrix properties and does not adequately capture intracellular contributions or cell membrane integrity. Bioelectrical impedance spectroscopy, which spans a broader frequency range, has been shown to more accurately separate intra- and extracellular fluid contributions and provide deeper insights into tissue physiology [28]. Thus, although the present study demonstrates that single-frequency BIP features can yield clinically useful discrimination and prediction, their biological interpretability remains limited.

Third, since the dataset was obtained from our previous study, key confounding variables known to influence bioimpedance measurements (e.g., hydration status, time of day, recent activity, skin temperature, medication) were not explicitly controlled. In that protocol, measurements were performed under standardized conditions, including a resting period before assessment, alcohol cleansing of the skin, and ultrasound gel application to minimize skin–electrode variability. While systemic factors such as hydration status may still have contributed to residual variability, the previous study nevertheless demonstrated robust discrimination between the LBP group and the healthy group using BIP indices, with ROC-AUC values of 0.984 for R, 0.984 for Z, and 0.963 for PA, alongside sensitivities above 0.92 and specificities above 0.93. These findings indicate that although such confounders warrant consideration, they did not overwhelm the pain-related signal in practice. Nevertheless, future studies should incorporate stricter protocol control.

Finally, participant recruitment in the prior study did not document pain duration (acute vs. chronic) or pain type (specific vs. nonspecific) As a result, stratification by clinically important subgroups was not possible in the present analysis. Although this limitation was already noted in the previous study [8], it remains an important consideration for interpreting the current findings. Future studies should therefore incorporate more detailed recruitment criteria to enable subgroup-specific analyses of bioimpedance features.

Although fold-wise uncertainty bands indicate robust internal performance, generalizability must be established on independent sites/devices. Future work should pursue multi-center external validation and calibration; incorporate multi-frequency/spectral BIP and segmental mapping; stratify acute vs. chronic LBP; evaluate fairness across sex/age/BMI subgroups; and integrate complementary modalities (e.g., imaging, wearable activity, psychosocial scales) to strengthen ODI/RMDQ prediction.

## 5. Conclusions

In this study, we applied an explainable machine learning framework to BIP collected in the lumbar paraspinal region. Our findings demonstrate that XGBoost models combining BIP features with demographic variables achieve strong discrimination between individuals with LBP and healthy controls, underscoring the clinical relevance of impedance-based biomarkers for objective pain assessment. In terms of pain score prediction, the models could provide clinically interpretable estimates of VAS scores, whereas predictive power for ODI and RMDQ was limited, reflecting the greater psychosocial influence of the latter scales. SHAP analysis further identified physiologically plausible predictors, such as PA and waist circumference, lending interpretability to the models and supporting their mechanistic validity. Together, these results extend prior correlation-based studies by showing that machine learning can extract clinically meaningful information from BIP data. This contributes to the growing evidence base for objective assessment of LBP, highlights the potential utility of impedance-based ML tools in clinical practice, and suggests directions for future research to refine prediction of functional disability outcomes.

## Figures and Tables

**Table 1:** Anthropometric/demographic and clinical characteristics of the participants.

Variable	Healthy (*n* = 45)	LBP (*n* = 38)	*p*-Value
Height (cm)	167.6 ± 9.0	163.8 ± 9.4	0.061
Weight (kg)	63.1 ± 13.0	66.2 ± 11.6	0.258
Waist circumference (cm)	77.0 ± 10.4	89.0 ± 8.2	<0.001 ***
Spinal length (cm)	3.99 ± 1.04	3.99 ± 0.78	0.976
Sex (M/F)	19/26	22/16	0.335
ODI score	5.4 ± 6.0	29.8 ± 11.3	<0.001 ***
RMDQ score	0.6 ± 0.9	6.6 ± 5.3	<0.001 ***
VAS score	0.38 ± 0.72	6.18 ± 1.52	<0.001 ***

Independent *t*-test was used for continuous variables and chi-square test for categorical variables. *** *p* < 0.001.

**Table 2:** Permittivity and sex-based tissue ratios.

Tissue Type	$\mathrm{Permittivity}\;(\varepsilon_{r}$) at 182 Hz	Ratio (Male, %)	Ratio (Female, %)
Skin	$1.14\times 10^{3}$	15	15
Fat	$7.20\times 10^{4}$	20	30
Muscle	$4.22\times 10^{6}$	42	38
Blood	$5.26\times 10^{3}$	8	8
Bone	$7.82\times 10^{4}$	7	7
Connective tissue	$6.11\times 10^{6}$	8	2

**Table 3:** Feature list.

Feature	Feature List
Demographic	Height, weight, sex (male = 1, female = 0), waist circumference, and spinal length
Bioelectrical impedance	R_right, Z_right, PA_right, C_right, R_left, Z_left, PA_left, and C_left

**Table 4:** Optimal hyperparameter using Bayesian optimization.

Parameter	Value
n_estimators	409
max_depth	5
learning_rate	0.162
subsample	0.867
colsample_bytree	0.607
gamma	4.478
reg_lambda	0.060
reg_alpha	0.012
tree_method	hist

**Table 5:** 5-fold cross-validated performance of the tuned XGBoost classifier across feature groups.

	Accuracy	Precision	Recall	Specificity	F1-score	ROC_AUC
Demographic	0.733 ± 0.103	0.730 ± 0.133	0.717 ± 0.240	0.755 ± 0.182	0.698 ± 0.143	0.765 ± 0.083
BIP	0.964 ± 0.052	0.975 ± 0.056	0.950 ± 0.068	0.977 ± 0.049	0.961 ± 0.056	0.986 ± 0.031
All	0.952 ± 0.049	0.975 ± 0.055	0.925 ± 0.068	0.977 ± 0.049	0.948 ± 0.052	0.996 ± 0.009

“Demographic” includes sex, height, weight, waist circumference, and spinal length; “BIP” includes bilateral R, Z, PA, and C; “All” combines both sets.

**Table 6:** VAS, ODI, RMDQ score prediction results.

	MAE	RMSE	R^2^	Spearman R
VAS	1.229 ± 0.268	1.641 ± 0.414	0.702 ± 0.140	0.787 ± 0.082
ODI	8.975 ± 1.311	11.312 ± 2.001	0.330 ± 0.377	0.650 ± 0.141
RMDQ	3.229 ± 0.626	4.637 ± 1.087	−0.087 ± 0.373	0.585 ± 0.232

**Table 7:** Performance comparison of ML models for LBP classification.

	Accuracy	Precision	Recall	Specificity	F1-Score	ROC_AUC
XGBoost	0.952 ± 0.049	0.975 ± 0.055	0.925 ± 0.068	0.977 ± 0.049	0.948 ± 0.052	0.996 ± 0.009
LR	0.976 ± 0.05	0.975 ± 0.05	0.975 ± 0.05	0.977 ± 0.05	0.975 ± 0.05	0.972 ± 0.00
RF	0.964 ± 0.05	0.975 ± 0.05	0.951 ± 0.06	0.977 ± 0.05	0.961 ± 0.05	0.944 ± 0.01
SVM	0.964 ± 0.06	0.9528 ± 0.06	0.975 ± 0.05	0.955 ± 0.03	0.961 ± 0.03	0.944 ± 0.01

## Data Availability

The data presented in this study are available on request from the corresponding author due to privacy restrictions.
